# Organizing the Methodological Toolbox: Lessons Learned From Implementing Developmental Methods Online

**DOI:** 10.3389/fpsyg.2021.702710

**Published:** 2021-09-13

**Authors:** Jonathan F. Kominsky, Katarina Begus, Ilona Bass, Joseph Colantonio, Julia A. Leonard, Allyson P. Mackey, Elizabeth Bonawitz

**Affiliations:** ^1^Graduate School of Education, Harvard University, Cambridge, MA, United States; ^2^Department of Psychology, Rutgers University, Newark, NJ, United States; ^3^Department of Psychology, Harvard University, Cambridge, MA, United States; ^4^Department of Psychology, University of Pennsylvania, Philadelphia, PA, United States; ^5^Department of Psychology, Yale University, New Haven, CT, United States

**Keywords:** developmental psychology, online studies, metascience, behavioral methods, infant, early childhood

## Abstract

Adapting studies typically run in the lab, preschool, or museum to online data collection presents a variety of challenges. The solutions to those challenges depend heavily on the specific questions pursued, the methods used, and the constraints imposed by available technology. We present a partial sample of solutions, discussing approaches we have developed for adapting studies targeting a range of different developmental populations, from infants to school-aged children, and utilizing various online methods such as high-framerate video presentation, having participants interact with a display on their own computer, having the experimenter interact with both the participant and an actor, recording free-play with physical objects, recording infant looking times both offline and live, and more. We also raise issues and solutions regarding recruitment and representativeness in online samples. By identifying the concrete needs of a given approach, tools that meet each of those individual needs, and interfaces between those tools, we have been able to implement many (but not all) of our studies using online data collection during the COVID-19 pandemic. This systematic review aligning available tools and approaches with different methods can inform the design of future studies, in and outside of the lab.

## Introduction

In many ways, the COVID-19 pandemic has accelerated technological trends in psychological research, such as the use of online data platforms to carry out “research at scale.” Developmental research has tended to lag behind in adopting these alternatives, likely due to the demanding methodological sensitivities required for child participants. Nonetheless, the health-safety issues of the past year have forced developmentalists to confront these methodological challenges and consider safer alternatives to in-person studies. This has revealed myriad potential advantages to online developmental research. Online research may enable labs to recruit more diverse samples, reduce barriers for participation compared to coming into the lab, facilitate longitudinal research by allowing for easier repeated access to the same participants, save researcher time by automating data collection, allow for naturalistic data collection, and more (Sheskin et al., 2020; see also Lourenco and Tasimi, 2020). Thus, there is ample reason to continue conducting developmental research online even after the COVID-19 pandemic has passed. The focus in this paper is to highlight the methodological lessons of this past year, to create a framework to help other researchers understand their methodological needs, and to identify available solutions for running developmental studies online.

Our methodological experiences are not necessarily novel. In the years leading up to the COVID-19 pandemic, a handful of developmental researchers were already pioneering various techniques for running experiments with children over the internet, without having to bring them into the lab (e.g., Scott and Schulz, 2017; Sheskin and Keil, 2018; Rhodes et al., 2020). However, once the pandemic hit, in addition to existing tools and techniques being used much more heavily, a number of new tools and techniques were quickly devised and put into practice. Because of the speed and urgency of this development, there are few compilations of the different techniques that different labs came up with, or the rationales behind why different techniques were used. To help researchers identify the best tools to conduct their developmental research online, we focus on a framework that starts with identifying the methodological constraints of a specific study, and we then present the available tools that meet those constraints. In addition, we consider the potential limitations or issues that these different approaches introduce and suggest ways to address those problems. We also discuss issues with recruitment and data quality that may arise with different approaches. In this way we hope to ‘organize the methodological toolbox,’ providing an easy reference for researchers to use when designing new studies in order to figure out how best to implement a given study online. The goal of this particular manuscript is to provide a how-to guide, rather than a comprehensive comparison between online and in-person methods (though we believe such comparisons should be a high priority for research in the coming years).

In the first part of this paper, the authors present six case studies from our own research methodologies, in order to give a general sense of the different kinds of approaches that are available, and the different kinds of studies that can be run. These case studies cover a wide range of approaches, from a very direct translation of an in-person task to online, to studies that allow the experimenter to take advantage of the unique properties of videoconferencing, to studies where there is no experimenter at all, and data collection is fully automated. In each case, we describe the goals and measures the study used, the methodological constraints and the approach used to meet those constraints, and any notable problems that needed to be addressed during the study. Furthermore, we have collected examples and guides of each of the approaches used in these case studies in an OSF repository^1^, to provide concrete examples for researchers interested in using these techniques in their own research. While each of these case studies comes from investigations of cognitive development, the techniques described may be generally applicable to many areas of developmental research. In the second part, we abstract away from these case studies in order to examine different methodological constraints that might arise in the design of a developmental study, and specific solutions that are available to address those constraints, with special attention to the pros and cons of different approaches. We also briefly consider issues related to the demographics of online populations and barriers to participation, although these issues have already received far more extensive consideration in other work (Lourenco and Tasimi, 2020; Sheskin et al., 2020).

## Case Studies

### Case Study 1: Direct Translation of an In-Person Study to Online

This project (Kominsky et al., 2021b) started before the pandemic and was adapted for online data collection. In person, the project involved showing participants (4-year-olds) a Qualtrics (2005) survey loaded onto a tablet. In the survey, participants first saw a short training about what an “x-ray” was, and then were shown two videos. In one video, a fur- or feather-covered puppet moved back and forth across a stage in an apparently self-propelled manner. In the second video, the other puppet (whichever was not in the first video) was shown sitting in a pink tray, being moved back and forth across the stage. It was important, particularly in the self-propelled case, that the movement appear smooth and not jerky. After each video, participants were asked to choose which of three images showed the “insides” of that puppet. In-person, they simply tapped the image on the tablet.

It was possible to directly translate this study to online data collection, with only two major methodological constraints. First, we needed a way to implement the multiple-choice response method. Second, we needed a way to present the videos such that the movement of the objects would look smooth. The multiple-choice response method was straightforward. An existing solution from the Yale Cognition and Development Lab is perfect for this kind of paradigm: simply present each of the options on a different color background, and train participants to respond by naming the color of their choice (Sheskin and Keil, 2018). We had used this technique in an earlier project that was also run online, prior to the pandemic (Kominsky et al., 2021a). This response method avoids the problem of trying to decipher where children are pointing through a webcam, or figuring out a way to let them interactively click on a choice. This approach proved to be highly effective in this case: every one of the 30 participants run using this online method provided usable data.

For the video presentation, we found that Zoom screen-sharing was simply inadequate. At the start of the pandemic in particular, before the platforms underwent a substantial amount of development, screen-sharing had a framerate around 10 fps, and the graphical quality was such that the fur and feather textures of the objects became amorphous blobs of color. In order to present the stimuli smoothly and in high visual detail, we needed a solution that did not stream them from the experimenter’s computer, but instead downloaded them directly onto the participant’s computer, while allowing the experimenter to control when they were presented. The only system we found for this that could work on any computer operating system was a website called Slides.com, which allows you to create a slide-show, send the participant (or audience) a link, and then as you advance through the slides from the presenter account, the slides advance in the audience’s web browser as well. Videos are presented as HTML5 video tags, which are downloaded in the participant’s web browser and rendered on their own computer, meaning the video plays at its native resolution and framerate. slides.com is also free in a limited capacity, and a relatively inexpensive $7/month if you need to store more presentations or use certain advanced features, but we have found that the free account provides all the functionality required. The only notable downside is that it was impossible to truly randomize the order of presentation of either the choices or the trials. Rather, we had to manually construct multiple pre-randomized orders in new slide decks and assign participants to them in advance of starting the online session.

### Case Study 2: Processed Video Feed Over Zoom With Open Broadcaster Software

In another lab study investigating young children’s social inferences, we wanted to know whether 6- to 8-year-olds would calibrate decisions selecting from recommended tasks based on an instructor’s (false) beliefs about their competence (Bass et al., 2021). To this end, we designed an experiment in which a confederate (the “Teacher”) overestimated, underestimated, or accurately represented participants’ performance on a picture-matching game (between subjects). Using her “prior knowledge” of the participant’s ability, this Teacher then presented three new matching games and evaluated them as much too difficult, not difficult enough, or just right for the participant; children then ranked their preferences for which of these new games they want to play. Children’s verbal responses were coded into a spreadsheet by an experimenter in real-time, and this coding was checked with video recordings of the Zoom call by an independent coder after the study session.

Because this task would necessarily involve multiple testers (the experimenter and the ‘‘Teacher’’ confederate), coordinating schedules and technical setups would be difficult. Further, these studies must be carefully controlled across conditions, such that any experimental manipulations are delivered in exactly the same way every time, with no possibility for bias. To circumvent these potential issues, we instead opted to have only one live experimenter administer the task; we used pre-recorded videos of the Teacher to present to participants during the experiment, under the pretense that she was actually live in the call. The key piece of software used for this study was a program called OBS, or ‘‘Open Broadcaster Software^2^.” Using OBS, we were able to create a processed video feed that incorporated the experimenter’s webcam and pre-recorded videos of the Teacher. This approach is less bandwidth-reliant than screen-sharing, allowing for higher framerate and resolution. By presenting this video feed over Zoom (along with some carefully timed acting from the experimenter “in response” to the pre-recorded Teacher), we created the illusion that the Teacher was also live on the call and interacting with the experimenter. (For a demonstration of how to execute the acting and timing as the experimenter, see: https://osf.io/3r5cj/. For a full video of a child being run in this task, and an example of what this set-up ultimately looks like to the participant, see: https://osf.io/a4be7. For a tutorial on how to use this set-up, see: https://osf.io/8ycnf/.) Importantly, the task itself was quite complex for children: It involved recursive mental state reasoning (i.e., “I know that you know that I know…”), contextualizing pedagogical actions given a second-order false belief, and calibrating subsequent decision-making to that false belief. Nevertheless, children appeared to be sensitive to our experimental manipulation, even using this online, pre-recorded paradigm. Only three (out of 60) children’s data had to be dropped and replaced: two for failure to pass built-in memory checks, and one for terminating the task early. No data were dropped due to technical difficulties.

The prospect of being able to run such nuanced social cognitive reasoning tasks online is an exciting one, but there are also limitations to this approach. First, running these studies smoothly puts non-trivial hardware demands on the experimenter: We have found that the minimum specifications require a 2.3 GHz dual-core Intel i5 CPU and 16 GB of RAM. Second, there is a significant amount of preparatory work involved in recording and editing the pre-recorded videos, and in setting up the stimuli in OBS. (For the stimuli used in the OBS ‘‘scenes’’ for this study^3^ > Processed video feed over Zoom with OBS > Calibration to Teachers’ Knowledge > OBS scenes - materials.) Third, all experimenters need to be quite comfortable with acting. For instance, the timing required to make the ‘‘conversation’’ between the Teacher and the experimenter convincing was quite precise; and without the use of additional plugins (e.g., Voicemeeter Potato^4^), the experimenter is actually unable to hear any audio from the videos played through OBS, making this timing even more difficult. We also had to explain away the Teacher’s inability to interact with the participant; therefore, the experimenter and the Teacher had to feign surprise at an “unexpected technical glitch” that supposedly prevented the Teacher from being able to hear the participant. Indeed, this raises another perhaps obvious limitation of this approach: The pre-recorded actor cannot respond live to participants, which may decrease believability that the Teacher is actually live in the call. Finally, this task is quite long: Under ideal circumstances, it takes about 20 min, but it often runs longer than this. In addition to typical reasons that an online study might run long (e.g., parents requiring extra time to ensure Zoom is set up correctly), in this study in particular, children would often engage the experimenter with thoughts about how to “fix” the Teacher’s audio glitch so that they could correct her false belief about their competence, lengthening the overall time it took to administer the task. Armed with knowledge of these limitations, however, we believe this online approach represents a promising way of assessing children’s social cognitive development, even when experimental manipulations are quite subtle and task complexity is high.

### Case Study 3: Remote Investigation of Curious Play

Many studies in our lab require measuring children’s autonomous play with toys. One such approach, the “Novel Apothecary Box” task, was designed to measure 4- to 8-year-old children’s curiosity through their playful exploration of a box with many possible drawers (each containing unknown items). Specifically, the design of this task aims to quantify children’s exploratory behaviors similar to past studies of novel toy exploration (e.g., Bonawitz et al., 2011) via their discovery of a (bounded) set of unknown options, objects, or functions, in a naturalistic, play-like scenario.

The main challenge in designing the Novel Apothecary Box was determining how to emulate children’s experience of naturalistic play with toys. We were concerned that tablet-based interactions would not capture the life-like, proprioceptive experience of play, but also concerned about the feasibility and health risks of mailing a large toy to families and requesting return of materials following completion of the study. We thus devised a modified Apothecary task, in which participating families are mailed a ‘‘cheaper version’’ of our task, which they are then able to keep as thanks for participating in the study. Families received a package with sixteen envelopes containing different kinds of enclosed (inexpensive) small toys and play materials, split up into four color-coded categories of related items (e.g., magnetic items in the blue envelopes; pretend-play items in the yellow envelopes^5^). This package also contains written instructions for the child’s caregiver^6^, describing the purpose of the package and its contents for the family while also prompting them to wait for further instructions from the experimenters before opening envelopes or showing materials to the child participant.

The procedure itself is administered over video call (e.g., Zoom) with a live experimenter. After the experimenter ensures that the child’s webcam adequately captures the child’s hands and a playspace surface (approximately four-to-six square feet on the floor), the experimenter provides a prompt, highlighting one of the four color sets. Then, the child is free to explore the envelopes (and their contents) for up to 6 min, or until they notify the experimenter that they are finished playing. Play sessions are video recorded through the video call software.

From these video recordings, various aspects of children’s play are coded, including important dependent variables as measured in past studies on exploratory play such as: the amount of time children play with the envelopes and their contents, the amount of time children spend playing specifically with the demonstrated (blue) category, the number of envelopes children open (total and per color), the order in which children open and interact with each envelope and their contents, and the number of unique combinations of objects children try during their play. Importantly, this task design allowed for finer control of potentially important perceptual aspects of the stimuli that may otherwise be lost (or face noisiness) due to differences in available technology and devices on the participant’s end (e.g., device brightness, volume, on-screen object sizes, potentially undisclosed device damage).

Limitations of the apothecary task come about due to its nature as a play experiment-at-scale. Pre-pandemic, exploratory play studies used to measure children’s playful curiosity may have only required the creation of two sets of stimuli (e.g., one novel toy for testing, a second identical novel toy as a backup). Given that each participating family requires identical stimuli to maintain control over the experiment, the number of stimuli sets scales linearly with the number of participating families. For example, across our various studies employing this method, we have mailed more than 200 identical packages that must be purchased (∼$5 cost of toys items), hand-packed by participating experimenters, and mailed to families (∼$5 shipping). To mitigate the required labor in preparing packages, the items and packing materials were chosen in their simplest forms (single items in single envelopes) and prepared in large batches (typically up to 20 packages per batch). Additionally, depending on the climates of the locations between the experimenters and participants, issues with postal services may arise. Currently, we have only experienced approximately 10 percent attrition (103 of 114 recruited participants provided usable data) in regard to families not receiving the Apothecary task stimuli in the mail. For those who failed to receive their package, another package would be promptly prepared and sent to the participating family. Furthermore, if the experimental session were scheduled with an expected delivery date that was not met, participating families would simply be rescheduled to a future time slot, if desired.

### Case Study 4: Online Infant Habituation Studies Using PyHab

Experiments with infants and toddlers involve methods that mitigate developmental limitations on talking and acting. In a habituation study, both the order of trials and (typically) each individual trial are gaze-contingent (Colombo and Mitchell, 2009). A typical habituation study involves trials that end when the infant has looked at the stimulus for some amount of time, and then looked away for some amount of time or a maximum trial length has been reached. Habituation trials are presented repeatedly until a habituation criterion is met, typically something like a total gaze-on time during the most recent X trials that is some fraction of the gaze-on time in the first Y trials. This means that infants’ gaze behavior must be coded by the experimenter in real time, so the experimenter can end a trial at an appropriate time, present the next trial, and determine when to proceed from habituation to test trials.

We were conducting a habituation study with 6- to 7-month-old infants in which the stimuli required smooth framerates, and the procedure required live gaze coding in order to determine when infants were habituated and when each trial should end. In the lab, these studies were run with PyHab (Kominsky, 2019), an add-on for PsychoPy (Peirce et al., 2019). To adapt them for online use, we took advantage of PyHab’s open-source nature and modified it such that we were able to integrate it with a solution used in Case Study 1 (above) for smooth remote stimulus presentation: slides.com. In short, this modified version of PyHab controls a Slides.com presentation instead of directly presenting videos as it does in the lab. The parent of the participant is asked to open the slides.com presentation in a web browser and make it full-screen, so it is the only thing the infant can see, and then sit in such a way that the infant is visible on the webcam in Zoom. The experimenter then mutes themselves and watches the infant through the Zoom call, live coding whether the infant is looking at the screen or not, and PyHab determines when to end a trial and when to advance from habituation to test.

In many regards, once configured, the methodological experience is almost identical to running a habituation study in the lab, particularly if the experimenter is already familiar with using PyHab for in-lab studies. The initial setup is very different, however, and does require a small degree of technical skill to modify PsychoPy to interface with a web browser. We created a detailed step-by-step setup guide to help researchers do this setup more easily. This guide can be found at https://osf.io/g42rw/. In data collection to date we have had to exclude 2 of 17 participants, both due to environmental distractions (pets or siblings). Additional concerns regarding camera placement, home-based testing environments, and parental interference are discussed below.

### Case Study 5: Unmoderated Online Study of Toddlers’ Predictive Looks

Additional studies in our labs involve measuring infant looking behavior using eye-tracking and measuring concurrent brain activity, using EEG (electroencephalogram, measuring electrical activity recorded on the scalp, using specialized “nets” and software.) One such study was started before the pandemic, and originally involved EEG and eye tracking measures. Although it was impossible to move to an “online EEG” set-up, one aspect of the study could be salvaged. That is, one of the dependent variables of interest was whether toddlers would produce predictive saccades toward certain locations on the screen which would indicate that they have learned a rule. We define a ‘predictive look’ as an eye-movement toward specific locations on the screen, during a specific period of the trial, which is not elicited by any changes in the visual stimuli itself (the scene is static), but can be presumed to be driven by the participants’ expectation of how the events will unfold. Specifically, if participants learn that certain objects get placed in one location, and another type of objects in another location, we can test whether participants anticipate where an object will be placed, by examining whether they would saccade toward the correct locations, even when the placement does not in fact happen.

The study was adapted for online data collection using the platform Lookit (Scott and Schulz, 2017), developed by MIT Early Childhood Cognition Lab. Lookit offers experimenters a detailed tutorial and support on how to set up an online study, and offers participating families the possibility to take part in studies from home, at a time of their choosing, requiring only a computer device with a webcam. Participating in a study involves the caregiver reading or watching customized video instructions, created by the experimenter for the specific study, explaining the aim of the study, the duration, and the ideal set-up for optimal data collection. Video consent is obtained for each participant, and reviewed by the experimenter before access to the participant’s video recording is obtained.

Adapting an in-lab toddler experiment to an unmoderated online experiment introduces some challenges. Participants’ homes inevitably mean a less controlled environment for data collection than in-lab studies. In order to minimize the likelihood of losing data due to disruptions, poor video quality, or parental interference, detailed instructions with visual displays of how to participate are essential. Examples of video instructions for the participating families used in this study can be found here (https://osf.io/6f5dj/ and https://osf.io/9ep2t/).

Another issue is calibrating gaze position. The outcome measure of interest in this study is toddlers’ (15–18 months) predictive looks toward specific locations on screen. As opposed to in-lab studies – in which all participants would see the stimuli on the same screen, at the same distance, and positioned centrally with respect to the screen – self-administered online studies introduce variability in these parameters. To account for this variability and to maximize the reliability of analyzing toddlers’ looking behavior, we introduced ‘calibration’ videos immediately preceding each of the test videos. In these videos, a captivating animation is displayed against a black background, at each of the crucial parts of the screen sequentially (corresponding to the locations toward which predictive looks are expected). This was followed by a centrally displayed animation, to bring the toddlers’ attention back to the center of screen. These calibration videos allow the experimenter to establish what the participants’ eyes look like when they fixate each of crucial locations of the screen, and therefore facilitate accurate coding of predictive looks in the following test videos, even if the participant is not sat centrally or their head position is not upright and forward facing. An example of a calibration video (followed by test video) used in this study can be found here (https://osf.io/uvdjf/).

Finally, while in-lab equipment typically allows for combining video recording of the participant with the display of the stimuli that the participant is watching, Lookit recordings only include the video of the participant. This means that in order to track the progression of the stimuli that the participant is observing, the experimenter must rely on the audio of the recording. This poses a particular challenge, if the experiment involves many trials and the audio of the videos is identical across trials (as was the case in our study). Experimenters should be conscious of this constraint when designing an experiment and add audio cues (such as the calibration videos used before test trials in this study) to facilitate easier decoding of the recordings. Note that despite these precautions, some of the video recordings obtained through Lookit for this study did not contain the audio of the presented stimuli. It appears that certain webcams only record the audio coming from the environment, while filtering out the sound that is emitted by the device itself. This is an issue that, to the best of our knowledge, does not yet have a solution. It may therefore be good practice to design stimuli in ways that the illumination of the screen changes significantly (i.e., at the beginning of test trials), so that the reflection of this change may be detectable on the recordings of participants’ faces and used for coding.

### Case Study 6: Unmoderated Tablet-Based Game

This project was started before the pandemic. In its original form, children played with a physical wooden tree and used a pulley device to get an “egg” (a metal ball) back to a nest in the tree. Unbeknownst to the child, there was an electromagnet in the pulley device that allowed the experimenters to surreptitiously control when the egg fell off. We assigned children to conditions where the egg fell off at continuously closer positions to the nest or at about the same position each time. We were specifically interested in how children’s trajectory of past performance influenced their decision to keep playing with the current tree or switch to an easier, shorter tree.

We had the following criteria for a remote version of this study: (1) asynchronous data collection to avoid scheduling and internet issues, (2) interactive design where children could feel like they had agency over their play, and (3) parent supervision that could ensure data quality with 4--6-year-olds, but was not intrusive. Based on these criteria, we concluded that the best solution would be to build an interactive touch screen web-based game for children. A undergraduate research assistant with strong coding skills built the game with the JavaScript library React^7^, hosted on Heroku^8^, and used MondoDB^9^ for the database.

The web-based version of the game was fairly similar to the in-person version. Children still had to get an egg back to a tree, but this time used their finger to slide up the platform with the egg instead of using a physical pulley device. The egg wobbled as it went up and fell off at predetermined points. As in the in-person game, at the end, children chose whether to keep playing with the current tree or switch to an easier, smaller tree. We wanted parents to supervise their child’s play in case anything went wrong (child clicks wrong thing or closes game), but did not want parents to intervene. To this end, we instructed parents to quietly watch their child play and only answer questions in the game addressed to them (‘‘parent, please confirm that child pressed X’’). To ensure successful remote administration of the game, we had explicit instructions for the parent and child throughout. For example, we used pictures and verbal prompts to instruct children when to put their hands on their lap and listen and when to touch the screen and play. We also figured out that design features could serve as implicit instructions: we only displayed the egg on the screen when children were supposed to move it around. To make sure a child was playing the game, and not an adult, we audio recorded participants’ responses to questions about their name, age, and their final task choice using the npm module mic-recorder-to-mp3^10^. At the very end of the game, parents could also write in if there was any interference during game play.

We ran into three main issues with remote data collection: non-serious participants, game play issues, and voice recording problems. We had to halt our first round of data collection due to a large number of non-serious participants (∼40%). The non-serious participants were unintentionally recruited through our Facebook ads and we only started to see them after we increased our compensation from $5 to $10 in hopes of attracting more participants. We spotted them thanks to the audio recordings. It turns out it is easy to tell an adult voice from a child voice – two people listened to recordings and always agreed when someone was an adult. We stopped the non-serious participants from participating in our study by halting payment and not inviting participants to play who had questionable information in their sign-up forms (e.g., different dates of birth entered on separate pages of questionnaire). Another issue we experienced was children unintentionally closing or restarting the game part-way through play. Because the game requires moving a pointer finger up the screen, it was easy for children’s fingers to slip and refresh or close the page. Through the backend of the game, we received information on how many times children played the game and when they stopped playing. We usually followed up with parents directly via email to confirm details of their children’s game play if it was halted early. We ended up excluding children who did not play through the full game in one session on the first try (7% of recruited participants excluded for this reason). However, the largest contingent of participants we had to exclude were those who did not have audio recordings (11% of recruited participants). We are unsure of the reason behind this issue and are continuing to investigate solutions.

### Case Studies Summary

These six case studies illustrate a number of different approaches to conducting developmental research online, but this is far from a comprehensive list. Furthermore, the options that are available will certainly change as new technologies and services are developed. In the remainder of the paper, we will consider each of the columns in Tables 1A, B, including the factors that might go into each decision researchers can make in designing their experiment, and the tools that are available to experimenters based on those decisions. First, we examine issues of study design: how you construct your stimuli in the first place. Then we consider issues around actually running the study, i.e., the process of data collection. Finally, we discuss issues relating to the processing and analysis of data, including attrition, data reliability, and comparisons with in-lab data.

**TABLE 1A:** Features, advantages, and disadvantages of each of the six case studies.

	**Moderated**	**Stimulus fidelity**	**Setup effort**	**Technical requirements for implementation**	**Technical requirements to participate**
Case 1: Direct translation of in-lab study	Yes	High – framerate and visual quality are rendered by participant computer and comparable to in-lab	Moderate – Building multiple presentation orders in slides.com	Low – No specific technical skills required, just experience building powerpoint-like systems	Low – Parent only needs web browser
Case 2: Processed Video Feed over Zoom with OBS	Yes	High – framerate and visual quality are rendered by participant computer and comparable to in-lab	Moderate to high – Installing and configuring OBS and all necessary plugins, recording and editing videos, setting up scenes in OBS	Moderate to high – Smooth video presentation requires 2.3 GHz dual-core Intel i5 CPU and 16 GB of RAM	Low – Parent only needs Zoom, and can use any device with a sufficiently large screen to see stimuli (tablet, desktop, laptop, etc.)
Case 3: Remote investigation of curious play	Yes	High – Stimuli are physical objects in the real world	Low to moderate – Minimal technical requirements as noted alongside proper participant cooperation for camera setup	Low to moderate – Current stimuli sets requires a minimal understanding of electronics	Low to Moderate – For behavioral coding purposes, a stable internet connection is required
Case 4: Online infant habituation studies using PyHab	Yes	High – framerate and visual quality are rendered by participant computer and comparable to in-lab	High – Configuring PsychoPy, setting up the slide show, inputting stimulus information into PyHab experiment	Moderate to high – Setup requires modifying PsychoPy with additional libraries, running PsychoPy, Zoom, and a web browser simultaneously	Low – Parent only needs web browser
Case 5: Unmoderated online study of toddlers’ predictive looks	No	High – framerate and visual quality are rendered by participant computer and comparable to in-lab	High – arranging between-institution ethics agreement, coding the experiment on Lookit, recording and editing stimuli and instructions videos, peer review process of study.	Moderate – Setup requires video editing software for stimuli and instructions videos, and use of several online platforms – stimuli repository, experiment coding on Lookit, Slack for set-up support.	Low to moderate – Participation requires a device with a webcam and web browser, setting-up an account on Lookit, and following the set-up instructions and recording a video consent.
Case 6: Unmoderated tablet-based game	No	Medium – framerate and visual quality are rendered by participant computer, BUT dependent on participant’s internet connection	High – coded game in JavaScript, handled database with MondoDB	High – Setup requires coding in JavaScript and general coding knowledge.	Low – Parent only needs web browser and touchscreen device (tablet, phone)

*The first column is descriptive. The remaining columns offer the experimenter’s subjective opinion of different features of the methods.*

**TABLE 1B:** Features, advantages, and disadvantages of each of the six case studies with regard to data collection and analysis.

	**Running effort**	**Data processing effort**	**Attrition**	**Data reliability**	**Monetary cost**
Case 1: Direct translation of in-lab study	Moderate – Similar to running a study in the lab, but with a browser and a Zoom window	Moderate – Data must be recorded outside of the presentation system, either manually or by coding video	Low – no online participants had to be excluded in this study	High – no difference between an in-lab and online sample.	Moderate – participant compensation in gift cards, potentially slides.com subscription
Case 2: Processed Video Feed over Zoom with OBS	Moderate – Similar to running a study in the lab, but with OBS, a browser and a Zoom window	Low to moderate – Data are manually coded by experimenter in real time, and checked with video recordings by an independent coder after the study session	Low – No higher than in-lab studies	High – Behavioral data from both adults and children were in line with our predictions	Low to Moderate – participant compensation in gift cards (all software is free)
Case 3: Remote investigation of curious play	Moderate to High – Data collection entails creation of multiple sets of physical stimuli, scaling with the projected sample size	Moderate – Data must be recorded/coded by a condition-blind researcher after the post-data collection session	Low – Participants and their families typically prepare a testing space adequately to ensure data means quality standards needed	Moderate to High – Behavioral data following similar trend to in-person samples. However, data collection and analysis is ongoing.	Moderate to High – Costs include compensation to participants, materials and labor for preparing packages, and shipping fees for delivering packages to participants
Case 4: Online infant habituation studies using PyHab	Moderate – Similar to running a habituation study in the lab, but with Zoom and PyHab	Low – Data recorded automatically by PyHab	Low – no higher than in-lab, and we are getting fewer fuss-outs from 6–7-month-olds	Moderate to High – Trends and SDs are thus far similar to in-lab, but data collection is ongoing.	Moderate – participant compensation in gift cards, potentially slides.com subscription
Case 5: Unmoderated online study of toddlers’ predictive looks	None – data collected without involvement of experimenter.	Moderate – data is manually coded from video recordings by two independent and condition-blind researchers, post data-collection.	Moderate (∼30%) – some video recordings could not be reliably coded due to audio issues, and poor positioning/visibility of the participants’ eyes.	Moderate to High – prevalence of predictive looks similar to that found in-lab, using an eyetracker.	Low to Moderate – all online platforms are free to use. Experimenters can offer participating families compensation in the form of e-vouchers.
Case 6: Unmoderated tablet-based game	Low – data collection required emailing interested participants and paying them after participation	Moderate – voice recording data had to be manually checked and double entered. Confusing cases were discussed over email with parent.	Low to Moderate – excluded ∼20% of data collected for issues with game play, audio recordings, or incorrect age.	High – no difference between an in-lab and online sample.	Moderate – compensation in gift cards, potentially paying someone to program the task.

*Each column offers the experimenter’s subjective opinion of different features of the methods.*

## Designing a Procedure: What Do You Need to Do, and How Can You Do It?

**FIGURE 1:**
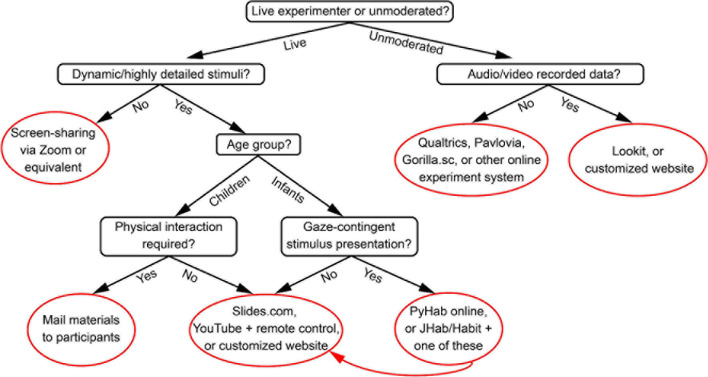
A quick-reference flow-chart for determining which types of paradigms might be appropriate for different study designs. See text for detailed considerations of each decision point and solution.

Here we will discuss different decisions researchers need to make in designing their studies. For easy reference for mapping decisions to tools, we provide a summary flow chart (Figure 1) which lists various solutions for different kinds of study design. However, in this section we also consider *why* you might choose to conduct a study in one way or another, to help researchers make informed decisions.

### Moderated vs. Unmoderated: Do You Need an Experimenter?

We have found one of the most foundational decisions about online study design is whether the experimenter needs to be present during the experiment (i.e., “moderated”) or if the experiment can be run completely automatically, generally through a website of some kind (i.e., “unmoderated”). For some types of studies, an experimenter is absolutely necessary, including studies that examines how children interact with an adult as a primary question of interest, or infant habituation studies that requires gaze-contingent stimulus presentation (at least until automated gaze-coding technology becomes substantially more advanced; Chouinard et al., 2018). For other types of studies, it is a choice, and there are advantages and disadvantages to each type of study.

For moderated studies like Case Studies 1–4, with a live experimenter, there are a number of clear upsides. First and foremost, a live experimenter can adapt better than an automated system to situations that might arise. An experimenter can ensure that data are being recorded correctly (e.g., the participant is visible on the webcam and can be heard), and the experimenter can be responsive to the participant’s behavior in order to keep them engaged with the task. This is also relevant for tasks in which there are follow-up questions that are contingent on what the participant says. For example, while most automated systems can have a branching task structure, at least with our current technology, it would be difficult to have an automated task that reliably responded to a *verbal* response made by a participant. Second, moderated designs are more comparable to most in-lab studies. While there is value in replicating a study that was previously run by a live experimenter using an automated system (e.g., Scott and Schulz, 2017), if you are attempting to build on a previous finding and want to stick as closely to its methods as possible, running it with a live experimenter may be preferable.

The downsides of having a live experimenter are primarily that it requires scheduling an appointment and takes the experimenter’s time. Unmoderated studies are completely on the participants’ schedule, while moderated ones require coordination between the participating family and the experimenter(s). Of course, that’s also true of in-lab studies, and in fact moderated online studies are much easier to schedule and run than in-lab studies because they don’t require anyone to travel. Furthermore, there are tools that can make signing up easier, such as using automated scheduling tools like Calendly or YouCanBookMe to allow parents to select a time that works for them, and these services often provide automated reminder emails that reduce no-shows. Anecdotally, we have found that an automated reminder email sent from the scheduling service 24 and 1 h before the appointment with a link to cancel or reschedule leads to very few unexpected no-shows (though we have not quantified the no-show rate precisely because people who do not show up for the study at all are not counted as participants). The other potential downside, which is again shared with typical in-lab studies, is that moderated studies introduce the possibility for experimenter effects or inconsistency between participants that unmoderated studies do not.

One of the advantages of unmoderated studies like Case Studies 5–6 is that, as mentioned, they do not require coordinating schedules between participant and experimenter. The participant can take part in the study at any time. Aside from just being easier, this also matters for studies that are trying to recruit from a global population: you don’t need to worry about time zones. It also places zero burden on the experimenter, other than advertising the study and dealing with the data. For researchers who work with adults, the difference between running a study in the lab and running it over MTurk and Prolific is hard to overstate. A study that would take weeks or months in the lab often takes no more than a day through large online collection sites, all while the experimenter can be working on other things. In our experience, you don’t typically get the same kind of pace of data collection with unmoderated developmental studies as you do with adult unmoderated online studies, but it is easier on the experimenter’s schedule.

There are several drawbacks to unmoderated developmental studies, however. First, much careful thought needs to be put into how they are set up. Depending on the nature of the study, it may also require substantial technical skills to set up, involving programming in JavaScript or even full web development. Furthermore, there is a real challenge in the “user experience” aspect of the study. The experimenter is not present to give instructions or correct anything the participant does, so the study must be thoughtfully designed to ensure that the participant completes the task as intended. Lookit (Scott and Schulz, 2017) has taken great pains to design rich instruction templates for this very reason (these are described in the Lookit tutorial^11^), but if you are not using an established system, you would need to design your own. The lack of a live experimenter also means you may face challenges with data quality and attrition (see the “Data: Attrition and Quality” section below).

Based on these considerations, researchers should think carefully about whether a study is better served being moderated or unmoderated. That decision then constrains which tools are appropriate for the study.

When considering tools for moderated studies, the solution is almost always going to involve some kind of video-conferencing software such as Zoom, Skype, FaceTime, Google Meet, Adobe Connect, or others. There are different stimulus presentation systems that can be used alongside the video-conferencing software, depending on the needs of the stimuli (see next section), but the video-conferencing software is always how the experimenter interacts with the participating family. In principle one could also run a study over a phone call or with mailed surveys, but it would be much more restricted in terms of the types of data that could be collected, and the types of stimuli that could be presented.

For unmoderated studies, there are a variety of potential solutions, but the first major consideration is what kind of data the researcher intends to collect. If video or audio recordings of the participant doing the study are needed, the available solutions are Lookit, or something custom-built that can access the participant’s webcam and/or microphone via the web browser (e.g., see Case 6 and the accompanying materials in the OSF repository at https://osf.io/rzh9d/). On the other hand, if it is sufficient to collect responses via keyboard, mouse, or touchscreen, there are several options. For simple survey-like studies that involve multiple choice elements, even with audio or video, there are services like Qualtrics, SurveyMonkey, and others. These systems often offer institutional licenses, so check with your university IT office about whether an online survey system is available to you. For studies that involve more complex stimuli or tasks, there are online psychophysical study presentation systems like PsychoPy’s Pavlovia, Gorilla.sc, Labvanced, jsPsych, Testable, OpenSesame, and others (for a careful examination of the stimulus presentation capabilities of many such systems, see Bridges et al., 2020). Of course, it is also possible to create a custom web app instead if the researcher has or has access to someone with the required technical skills. An additional option, which requires yet further technical skills and substantial effort, is to create a data collection platform for mobile devices (e.g., Kid Talk Scrapbook^12^). The use of mobile apps for developmental research is still new and relatively untested at time of writing, though there has been at least one pediatric medical study using a mobile app-based platform (Lalloo et al., 2020).

Finally, there is a sort of compromise category that is neither strictly moderated nor unmoderated: asking parents to serve as experimenters. Some unmoderated studies are effectively already like this, they ask the parent to monitor their child completing the task to keep them focused. In general, a study like this would share many of the advantages and disadvantages of an unmoderated study but might allow for some designs that would otherwise be impossible. For example, consider a study that focused on a particular daily routine and asked parents to record that routine, and ask specific questions during it, and then send those videos to the experimenter (Leonard et al., 2020). It is something of an edge case, but for certain research questions it may be the best approach.

### Stimulus Presentation: Speed and Detail

Stimulus presentation introduces another important consideration for developmental studies. Particularly for moderated studies, a key concern is how high-fidelity the stimuli need to be. First, let us carve out an exception: studies like Case 3, in which physical stimuli are delivered to the participating family, are obviously the highest possible level of fidelity. If the research question involves children physically interacting with an object, this is obviously necessary, but the cost and logistical difficulties introduced by shipping materials to each individual participant are high. This section will mostly be concerned with screen-based stimuli.

In terms of screen-based stimulus quality, there is one key technical consideration: is the experimenter’s computer rendering the stimuli and then streaming it to the participant’s computer via screen-sharing of some kind, or are the stimuli being rendered on the participants’ computers directly? Screen-sharing imposes some caps on the quality of stimuli in various ways. The resolution (number of pixels/level of detail) may be restricted, and for dynamic stimuli, the frame-rate may be reduced or unstable. Case 1, above, presents an example of stimuli that could not be used with screen-sharing. However, if the stimuli for an experiment are static images or otherwise do not lose relevant information if the video quality or frame-rate should happen to drop, there is no reason *not* to use screen-sharing. There are two advantages to screen-sharing over other solutions. First, it is the easiest way for the experimenter to control the stimulus presentation, because the stimuli are being displayed on the experimenter’s own computer and that view is being sent to the participant. Second, it can be easier for the participant (or their parent) to set up, because it does not require them to open a separate web browser or other program in order to view the stimuli, just the video-conference they would have to open anyways.

There are also multiple ways to stream stimuli from an experimenter’s computer over a video-conference, and different approaches can offer some methodological flexibility. For anyone who has used Zoom, the most obvious and simple solution is the built-in screen-sharing feature, and many other video-conferencing systems offer similar capabilities. In these cases, the image on the experimenter’s screen is captured by Zoom and transmitted to the participant alongside the image captured from the experimenter’s webcam. The frame-rate of screen-sharing like this is typically low, often capping out at 10–20 frames per second, subject to the upload speed of the experimenter’s internet connection *and* the download speed of the participant’s internet connection. An alternative solution is the one described in Case 2, in which the experimenter uses an additional program to create a processed video feed that is treated as a “virtual webcam.” This can sometimes offer slightly higher-quality video performance because only one video feed is being transmitted instead of two, so it is less restricted by upload speed. However, the main advantage is that it allows for designs like the one described in Case 2, in which there is a pre-recorded additional experimenter ‘present’ on the video call in a way that looks convincing, and does not require an additional experimenter to actually join the video call.

However, in cases where the stimuli need to be higher-quality, the best solutions are going to be those that download the stimuli on to the participant’s computer directly in some way and have the participant’s computer render the stimuli at their native resolution and framerate. Unmoderated studies necessarily do this: the participant accesses a website which downloads the stimuli into their browser and renders the stimulus file at its native resolution and frame-rate. There are various ways to achieve this in a moderated study as well, but it typically involves asking the participant to open a web browser and navigate to a particular website where the stimuli are hosted. In Cases 1 and 4 above we describe one such system, Slides.com, which has the dual advantages of allowing the experimenter to control when the stimuli are presented and of being platform-universal (i.e., not restricted to Windows or Mac systems). However, it has other limitations, notably an inability to keep the experimenter blinded to the experimental condition or randomize presentation order on its own (though Case 4 works around this by having PyHab control the order of slides).

Another solution some researchers have used is asking participants to share *their* screen with the experimenter and using Zoom’s ‘remote control’ function to allow the *experimenter* to control the stimuli *on the participant’s computer* (Liu, 2020). Essentially, the participant hands over partial control of their computer to the experimenter. Alternatively, the experimenter can send the participant to the sort of website that would host an unmoderated study, like a Qualtrics survey or even a Pavlovia (or equivalent) experiment, and have them complete the study while talking to the experimenter. This provides a way to conduct an interactive task (i.e., in which the participant directly interacts with objects on the screen) with the advantages of a moderated study. Across all of these solutions, it is often worth asking the participant to share *their* screen with the *experimenter*, so that the experimenter can record what the participant is seeing. In Zoom, this also keeps the experimenter visible as a small window in the corner but allows the stimuli to take up the bulk of the screen.

One concern that can arise using systems that present stimuli through a web browser is whether the stimulus files are in a format that will work on the participant’s computer. When screen-sharing, as long as the stimuli render on the experimenter’s computer, they are fine, because what appears on the experimenter’s screen is what the participant will see. For other presentation systems, the safest thing to do is use universal file formats. The safest file format to use is MPEG-4 (.mp4) made with h.264 compression, because these types of video files are supported by all major web browsers and operating systems as of 2021. For audio files, .wav files are safely universal, as are .mp3 files, though *creating* .mp3 files can be more difficult because it is a proprietary codec. In terms of making the stimulus files themselves, whatever solutions researchers have used in the past should still work, provided they can export to these standard file formats (and most audio and video editing software can do exactly that).

To sum up, what kind of solution researchers should use will depend on the level of visual quality your study requires, the nature of the stimuli, the level of interactivity required, and what solutions the researchers are most comfortable with from a technical perspective. In Figure 1, we summarize these considerations in what we hope will prove an easy reference for researchers figuring out what kind of tools to use for their online studies.

## Running Studies and Dealing With Data

Developmental studies must be sensitive to the abilities and nature of their participants. It would not make sense to design a study for 6-month-olds that required a verbal response, for example, or a study for 3-year-olds that required attending to a tedious task for 30 min. This is obviously still true when it comes to online studies, but there is an additional constraint that researchers should consider: the technical demands on the participant and their parents to participate in the first place. In general, researchers should strive to make an online study as easy as possible for participants to take part in. In other words, as much as possible, participating in an experiment should not require participants or their parents to need to conduct extensive technical setup, rely on parents using a specific operating system or web browser, or reconfigure the space in their home in which the experiment will be run. There are some specific cases where some of these might be unavoidable, for example a study that involved examining toddler’s mobility behavior at home would require there be a sufficiently large space for them to move around in, but in general we should strive to make the barriers to participation as low as possible, especially given that merely having a computer, reliable internet connection, and time can all be barriers to many participants (Lourenco and Tasimi, 2020).

Of the solutions discussed in the previous section, none require the installation of specific software on the participant’s computer beyond a web browser and video-conferencing software (which in many cases can run through a web browser anyways). It is our opinion that Lookit (Scott and Schulz, 2017) demonstrates a reasonable upper limit of what we can ask of parents, particularly for unmoderated studies, and Lookit asks as little as it can while still collecting usable data. The designers of Lookit have very carefully created a process that balances the demands on parents with the needs of experimenters. Participating in a study on Lookit requires no additional software or technology, but involves a multi-step setup in which parents are carefully walked through making sure their webcam and microphone are operative, recording a consent statement and test video, and making sure the participating child or infant is properly located on the screen. This step-by-step guide is the absolute minimum that can be asked of parents to ensure they will be able to complete a Lookit study successfully, and its instructions have been carefully refined over the years Lookit has been in operation. (see also the Lookit ‘getting started’ guide for more information about this process: https://lookit.readthedocs.io/en/develop/researchers-start-here.html).

There are some hardware constraints on the participant for these studies as well. The most obvious ones are a computer with a microphone and webcam. Some studies can be conducted on mobile devices like tablets or smartphones, but not all. For example, the techniques described in Case Studies 1 and 4 would not work on a tablet because most tablets cannot simultaneously run Zoom and a web browser, or they cut off the webcam when the web browser is the focal app. Studies that are run entirely in Zoom like Case Studies 2–3, or custom-programmed web apps like Case 6, could be run on a participant’s tablet or smartphone, at least in principle, though researchers should consider whether their particular study requires screens of a minimum size for effective stimulus presentation. At this time it is not possible to participate in a Lookit study (like Case 5) from a tablet, though future development could change that. More generally, depending on the nature of the stimuli and the study design, researchers should consider if there are minimum screen sizes or resolutions that would present difficulties. For perception studies that require more precise viewing conditions, there are techniques for asking participants to calibrate their screens using an object of standard size (for an example, see Bechlivanidis and Lagnado, 2016, Appendix A2). Even if that level of precision is not needed, it may be worth finding the most outdated computer and smallest screen at hand and seeing how, or even whether, it is possible to complete a new study on it before releasing it to the general public. More generally, experimenters should try to work out what minimum criteria need to be met for participants to take part in the study and include those in recruitment instructions.

Another factor to consider, particularly when designing a study for research assistants to run, is what demands your study places on the researcher. For example, Case 2 requires the experimenter to advance through a series of scenes in OBS while interacting with a child through Zoom, and timing those interactions such that the interactions with the pre-recorded stimuli presented through OBS are convincing. It is certainly doable, but it does require some practice! It also imposes some demands on the experimenter’s available hardware. We have found that older computers, particularly older Macs, have difficulty running both OBS and Zoom at the same time, and the video quality suffers heavily as a result. Particularly for studies that will be run by research assistants, it is important to ensure that those research assistants have access to adequate hardware to actually run the study. This is just one example, but in general, when designing an online study, researchers should consider how easy or difficult it will be for the experimenters to actually run with the required software.

One key design decision in terms of how difficult a study is for the experimenter is how the data are recorded. For unmoderated studies, the data, particularly audio and video data, will inevitably have to be coded offline by researchers. This is also an option for many moderated studies, particularly if the study is challenging to run already. For example, another study in one of our labs using the same approach as Case 2 was found to require so much attention by the experimenter just to execute that we elected to code all the data off-line rather than trying to note participants’ responses during the procedure. Some of the paradigms described above side-step this issue. For example, studies using PyHab, like Case 4, record data in the process of running the study with no additional effort. Of course, to ensure that the data are reliable, even in cases where the data are coded during the procedure, it is often worth having the data re-coded offline.

### Data: Attrition and Quality

While many of the case studies described above are still in process, we have collected enough data to examine attrition and more generally whether the data are of comparable quality to in-lab data, and in some cases how well the data align with results acquired in the lab. Case 1, for example, was run partially in person and partially online, and we conducted a comparison of the data collected online to the in-person data and found no reliable differences (Kominsky et al., 2021b). However, researchers cannot take for granted that this will be true for every study, and we have encountered different challenges in the different studies we have run.

Unmoderated studies face particular challenges, as noted above. When the experimenter is present, they can deal with obvious issues, for example ‘is there actually a child participating in this study.’ The study described in Case 6, in particular, ran into issues of non-serious participants (i.e., adults who took the study themselves just to get the participant compensation). As described above, when the project initially launched, the *majority* of participants were non-serious participants, and it was necessary to implement several types of screening to disincentivize these attempts to exploit the study for profit. Studies on Lookit, or other unmoderated studies that video-record participants, do not typically have this problem. A second issue is that the data itself is sometimes unusable for other reasons. For example, in the initial validation studies of Lookit, 35% of the videos recorded proved to be unusable due to some recordings failing for technical reasons, or the participant not being visible in the recording (Scott and Schulz, 2017). The technology has improved since then, but for any custom-designed solutions, extensive testing is needed to ensure that data are not lost due to technical issues, and piloting is strongly recommended to identify other potential problems in the data prior to opening the experiment to full data collection.

However, one note of caution for all online developmental research is that, relative to the history of our field, it is very, very new. We don’t know how comparable online data are to in-lab data for many paradigms, and there are very few systematic comparisons across in-lab and online data with children (Scott and Schulz, 2017; for work with adults see, e.g., Weigold et al., 2013; Hauser and Schwarz, 2016). The pandemic has likely created a number of ‘natural experiments’ like Case 1 (Kominsky et al., 2021b), i.e., studies that started in person and moved online, that may provide further insight on the matter as they are published (indeed, we suspect other papers in this collection may do exactly that).

## Closing Thoughts

In this paper we have attempted to provide a reference for researchers considering online developmental studies, to help them find the best tools and techniques for their particular needs. Broadly, by focusing on the key methodological constraints of a study, it can be relatively straightforward to identify the best tools for the job. There are some potential constraints we have not discussed in detail. Notably, the technical expertise available to the researcher can affect what solutions are actually achievable. Access to programming expertise, and particularly web development, can vastly expand the set of approaches available to a researcher, but these skills are not widely taught in our field. Universities sometimes offer such technological expertise to faculty in the form of dedicated research technology staff, but this is far from universal. However, most of the tools listed in this paper require no specific technical skills or programming ability, and were selected for this paper because they are accessible to researchers at any career stage and level of technical expertise. Furthermore, the majority of them are free, or at least inexpensive, and those that are not can often be licensed at the university level, making them affordable for individual researchers.

Evaluating the methodological constraints of a study and determining how to conduct it applies as much to in-lab research as it does to online research; in some cases the tools are even the same. PyHab (Kominsky, 2019) and PsychoPy (Peirce et al., 2019) are both designed for in-lab and online studies, and there’s no reason that any of the online presentation methods described here can’t be employed in the lab as well, and with a little more methodological flexibility. For example, Case 1 was originally conducted in-person using a Qualtrics survey presented on a tablet, because the experimenter could control advancement through the survey when they were physically present to click the ‘next’ button. Of course, in-person research also opens up a host of additional methodological possibilities, like neuroimaging, pupillometry, and eye-tracking, that simply can’t be done online with current tools. The methods that can be used for online research may also expand as new technology is developed: while it’s unlikely that we’ll ever be able to do remote fMRI, PET, MEG, or EEG studies, there is online eye-tracking for adult participants^13^, and developmental applications are currently under investigation, with some recent successes using offline analyses of videos to get fixation data (e.g., Chouinard et al., 2018; Chang et al., 2021).

Even when online studies are methodologically comparable to in-person data collection, they provide another source of participants that could perhaps represent a different population than is available in the lab, for better or worse. Thus, we come to a final point of consideration for all developmental research: participant recruitment. In-lab studies often recruit through databases of families that have expressed interest in participating in research, populated by purchased lists, state records, or other means. Of course, the families that actually participate are typically going to be those that are close to the lab itself, meaning that the demographics of a particular in-lab study will depend heavily on the lab’s location and the local populace, or at least on experimenters being able to physically port the “lab” to other locations. Museum-based studies face a different set of potential constraints, in some cases recruiting from a more representative population and in other cases recruiting from a narrower population that have the resources, time, and interest in visiting such locations (Callanan, 2012). For online studies, geography and (e.g.) admission fees are no longer relevant restrictions, but having reliable high-speed internet access may restrict the population in ways that have not been fully quantified (for further discussion of these issues see Lourenco and Tasimi, 2020). Different populations may also be more or less accessible by different recruitment approaches (e.g., advertising on Facebook or Google versus recruiting for online studies from an existing database). One promising recent development is a centralized website (like ChildrenHelpingScience.com or LookIt) for developmental researchers to advertise their studies to families, which allows all the labs using the website to benefit from each others’ recruitment practices, thereby potentially providing a much broader and more representative population than any one lab alone would be able to achieve (Sheskin et al., 2020). However, there are unavoidable minimum requirements for all of the online studies discussed here, and indeed nearly all online studies in principle: the participating family must have a device with internet access, a microphone, and in many cases a camera, that can be used in relative privacy, and researchers must take this limitation into account in interpreting their results.

Ultimately, once the COVID-19 pandemic has passed, the authors do expect to resume in-person data collection for many studies, but at the same time, we also expect to continue online data collection for others. For some designs, studies involving specific populations or specialized measures, in-person research will be preferable, but for others it may be easier or faster to continue to conduct research online. We believe this new wave of online developmental science will be long-lasting and bring many new benefits. Therefore, we expect that in the decades to come, developmental researchers will need to consider, for each new study, whether it is better to conduct it in person, online, or perhaps both at once, to make the most of all the methods that are now available to us.

## Author Contributions

JK wrote most of the original draft including two of the case studies, KB, IB, JC, and JL each wrote one of the case study sections. All authors participated in editing and revision.

## Conflict of Interest

The authors declare that the research was conducted in the absence of any commercial or financial relationships that could be construed as a potential conflict of interest.

## Publisher’s Note

All claims expressed in this article are solely those of the authors and do not necessarily represent those of their affiliated organizations, or those of the publisher, the editors and the reviewers. Any product that may be evaluated in this article, or claim that may be made by its manufacturer, is not guaranteed or endorsed by the publisher.

## Footnotes

1
https://osf.io/g42rw/

2
https://obsproject.com/

3
https://osf.io/rzh9d/files

4
https://www.vb-audio.com/Voicemeeter/potato.htm

5
https://osf.io/sc4rt/

6
https://osf.io/t89yp/

7
https://reactjs.org/

8
https://www.heroku.com/

9
https://www.mongodb.com/2

10
https://github.com/Hunterzhaoliu/learning_curve

11
https://lookit.readthedocs.io/en/develop/tutorial-access.html

12
https://www.kidtalkscrapbook.org/

13
http://turkergaze.cs.princeton.edu/
